# Building Immigration-Informed, Cross-Sector Coalitions: Findings from the Los Angeles County Health Equity for Immigrants Summit

**DOI:** 10.1089/heq.2019.0048

**Published:** 2019-08-23

**Authors:** Altaf Saadi, Mary L. Cheffers, Breena Taira, Rebecca Trotzky-Sirr, Parveen Parmar, Shamsher Samra, Janina L. Morrison, Sural Shah, Todd Schneberk

**Affiliations:** ^1^ National Clinician Scholars Program, University of California, Los Angeles, Los Angeles, California.; ^2^ Department of Emergency Medicine, LAC+USC Medical Center, Los Angeles, California.; ^3^ Department of Emergency Medicine, Olive View-UCLA Medical Center, Sylmar, California.; ^4^ Department of Emergency Medicine, Harbor-UCLA Medical Center, Torrance, California.; ^5^ Primary Care Internal Medicine, The Wellness Center, LAC+USC Medical Center, Los Angeles, California.; ^6^ Division of Internal Medicine-Pediatrics, University of California Los Angeles, Los Angeles, California.

**Keywords:** immigrant health, health disparities, public health, medical-legal partnerships

## Abstract

In December 2017, the Los Angeles County Office of Immigrant Affairs and Board of Supervisors, alongside local health care and legal providers, convened the Health Equity for Immigrants and Families Summit to advance a vision for immigrant health. We describe the four critical concepts identified by stakeholders to address the varied needs of immigrants in an increasingly anti-immigrant political environment: (1) Recognizing immigration status as a modifiable social determinant of health; (2) Adopting the concept of “Immigration-Informed Care” within health care institutions; (3) Establishing immigration-focused medical-legal partnerships; and (4) Building coordinated systems based on knowledge of local stakeholders, policies, and funding mechanisms.

## Introduction

Since the 2016 presidential election, anti-immigrant rhetoric and policies increasingly threaten immigrant communities in the United States.^1^ The hostile political climate is associated with decreased health care use and poor physical and mental health outcomes.^2–5^ Yet, the literature describing the impact of immigration status on health remains limited due to concerns for respondents' safety hindering data collection,^6^ and a paucity of practical strategies to improve immigrant health.

On December 15, 2017, the Los Angeles County (LAC) Office of Immigrant Affairs (OIA) and LAC Board of Supervisors, in conjunction with local health care and legal providers, convened the Health Equity for Immigrants and Families Summit.^7^ Attendees represented a broad range of disciplines, including legal experts, health care providers, administrators, health care enrollment and patient financial service providers, and community-based organizations and advocates. The summit highlighted cross-sector collaborations to address the needs of immigrant patients, irrespective of legal status, and identified strategies for effective multidisciplinary service delivery. Our goal is to share the wisdom of this unique stakeholder summit to guide other communities in serving this population.

## The LAC Context: The Leading Edge of Immigration Reform

Nationwide, 11 million undocumented immigrants reside in the U.S., nearly 10% of whom live in Southern California,^8^ including some of the longest-settled undocumented immigrants in the nation.^9^ In LAC, immigrants comprise nearly half of the workforce.^10^ Consequent to these county demographics and local community organizing, the elected officials, social service agencies, and legal and health care professionals are at the forefront of efforts to protect immigrants' rights. For example, in January 2017, the LAC Board of Supervisors created the OIA as a “one-stop shop” for immigrants to receive county assistance.^11^ In September 2017, the state legislature passed the California Values Act (SB 54) ensuring that local resources are not diverted to federal immigration enforcement efforts.^12^

Supported by this local political climate, stakeholders at the summit shared ideas and solidified a vision for advancing immigrant health. We describe the four key concepts derived from the summit:

(1) Recognize immigration status as a modifiable social determinant of health;
(2) Adopt the concept of “Immigration-Informed Care” within health care institutions;
(3) Establish immigration-focused medical-legal partnerships to improve immigrant health;
(4) Build coordinated systems based on knowledge of local stakeholders, policies, and funding mechanisms.

### Recognize immigration status as a modifiable social determinant of health

The World Health Organization defines social determinants of health as “conditions in which people are born, grow, live, work, and age… shaped by the distribution of money, power, and resources at global, national, and local levels, which are themselves influenced by policy choices”.^13^ Immigration status is unique in that it is influenced by social determinants of health (e.g., famine, war), influences social determinants of health after migration (e.g., working conditions, income inequalities), and is a social determinant in its own right that directly affects health (e.g., deportation, detention).

Although immigration status is increasingly recognized as a social determinant of health,^14,15^ it is incorrectly viewed as immutable. The idea that immigration status is not a static phenomenon, but rather a dynamic one with multiple pathways for legal status, represents a paradigm shift for health care professionals. Indeed, immigration status can be viewed potentially as dynamic as the acculturation process among immigrants, which can involve changes in socioeconomic status, language proficiency, or social networks. For example, an immigrant may enter the country on a valid visa, overstay his visa and become undocumented, apply for asylum and have a pending asylum application with potential for further review if denied, or subsequently obtain permanent residency status and citizenship. Health care professionals are poised to identify pathways to legal status during patient encounters, but these opportunities are frequently missed. For example, a patient may reveal they are the victim of a crime and have suffered mental or physical abuse. Consequently, they may be eligible for a U visa, a visa category set aside for victims of a serious crime, leading the path to legal status as a US resident and qualification for assistance programs. Similarly, others may be eligible for a T visa, which can be awarded to survivors of human trafficking.

Adjustment of immigration status leads to improved social conditions through better education, employment, and health care coverage. Disseminating knowledge to health care providers may not only improve health but also change that patient's life trajectory.

### Adopt the concept of “Immigration-Informed Care”

We propose the concept of “immigration-informed care,” building upon “trauma-informed care”,^16^ to describe health care settings that are primed with the knowledge and resources to meet the health needs of immigrants. Trauma-informed services encompass core principles of safety, trustworthiness and transparency, peer support, collaboration and mutuality, empowerment, and cultural competency and humility across all service providers, programs, and agencies.^17^ Trauma-informed services also utilize an intersectional approach that addresses the compounding impact of culture, history, race, gender, location, and language on trauma.^18,19^ As such, systems that incorporate a trauma-informed approach into their daily practice would offer services such as: routinely screening for trauma exposure, using evidence based and culturally responsive assessments and treatments for mental health symptoms, providing resources to families and clinicians on the treatment and impact of trauma exposure, engaging in efforts to strengthen the resilience and protective factors of children and families impacted by and vulnerable to trauma, emphasizing collaboration across service systems, and maintaining an environment that addresses secondary traumatic stress among staff members.

In addition to the core principles of trauma-informed care, relevant for this highly trauma-exposed population, components of “immigration-informed care” include appropriate language services, clearly delineated referral pathways for undocumented patients, culturally and structurally competent clinicians trained to discuss sensitive topics without inciting fear,^20,21^ and institutional policies that ensure the physical and psychological safety of immigrant patients, such as avoiding documentation of immigration status in medical records and limiting cooperation with law enforcement.^22,23^ Consequently, immigration-informed care would have a positive impact on patient care and patient–clinician partnerships as has the trauma-informed approach.^24^

Furthermore, an “immigration-informed” clinician understands migration as a continuum of experiences with a variety of stressors, including violence, sexual assault, and other trauma both in an individual's home country and during migration. Once in the U.S., barriers such as fear of discovery by immigration officials and mistrust of government-affiliated health services dissuade and prevent care-seeking behaviors.^25^ Many immigrants consequently rely on acute health care services. Furthermore, available resources are a patchwork of community and *ad hoc* efforts and may be difficult to navigate alone and in absence of social networks.

Backlogs in immigration courts result in a lengthy process that may last several years. Predatory, fraudulent legal schemes financially jeopardize families seeking assistance in navigating the legal process. Legal delays lead to years of limited health insurance eligibility, and cumulative mental health stress (summit proceedings). Deportation and detention, the risk of which is omnipresent, introduce additional stressors such as concerns about family reunification and disruption of the family unit or community structure.

### Establish immigration focused medical-legal partnerships

In recognizing immigration status as a modifiable social determinant of health, collaboration with the legal sector is imperative. Once a health care provider recognizes a patient who could benefit from a legal evaluation, the health care institution should have a referral pathway for legal assistance. Medical-legal partnerships (MLPs) are a prime strategy as they add lawyers to the health care team who specialize in addressing unmet social needs. With MLPs, legal barriers are considered central, rather than peripheral, to health concerns. Traditionally, MLPs include legal services related to receipt of public benefits, debt relief, or improved housing conditions.^26^ To address the needs of immigrant communities, however, these MLPs may include immigration legal services.

LAC launched an MLP in the LAC Department of Health Services (LAC DHS) in April 2017.^27^ In this MLP, trained community workers provide navigation services in the Emergency Department. If the patient navigator identifies a legal need, he/she connects the patient with an in-house immigration legal team to provide immigration status stabilization. This MLP currently receives on average 30 immigration referrals per month.

Legal integration with the health care teams addresses the shortage of immigration attorneys and the distrust that may result from immigration legal fraud. This fraud commonly consists of notaries posing as legal professionals who abscond with clients' payments. Immigrants with attorneys fare better at every stage of the legal process.^28^ In addition, legal providers not connected to a health care team struggle to provide appropriate medical referrals. Integrated MLPs, therefore, allow for bidirectional collaborations ideal for comprehensive patient care.

### Build coordinated systems on knowledge of local stakeholders, policies, and funding mechanisms

Immigrants are often unaware of their eligibility for health or social service programs and legal residence, further dissuaded to interface with legal and health sectors due to fear, distrust, and stigma. For example, some fear that enrollment into social service programs could make them ineligible for future citizenship—a fear heightened by recent proposed changes to the public charge rule.^29^

To address the disjointed patchwork of existing resources that leads to fragmented care, lapses in care, and redundancy, a comprehensive database of local knowledge and resources is a potential solution to enable cross-talk between sectors, institutions, professions, organizations, and individuals. Electronic platforms such as One Degree and Healthify are example platforms to support unification. Patient navigators and community health workers also strengthen referral conduits. Immigrants, particularly the undocumented, have fewer access points to care systems, making every service encounter an opportunity to build trust and make referrals on the patient's behalf.

Coupled with national policies are variations in local immigration-related policies that affect health care access. In California, state policies include AB 60, affording undocumented immigrants the opportunity to obtain a driver's license, and Permanent Residence Under Color of Law, allowing for Medi-Cal benefits in emergency and pregnancy-related situations. Systems must incorporate local policy variation, necessitating communication between medical providers, legal providers, and community-based organizations. Financial models must similarly incorporate local contexts. For example, a need for increasing capacity of immigration lawyers in Los Angeles led to suggestions about hospitals, including funding for legal services, recognizing addressing legal issues as vital to health.

## Conclusion

Promoting health equity includes responding to the unique health needs of immigrant populations. Immigration status is a modifiable, nonbinary social determinant of health with several possibilities for legal residence that are not citizenship. Unique programs such as the LAC immigration-focused MLP stabilize legal status to promote health. Health care institutions should adopt immigration-informed care and appropriately train providers. Coordinated systems, built with knowledge of local policy and funding contexts have the potential to improve health equity among immigrant populations.

## Abbreviations Used

LAC: Los Angeles County
MLP: medical-legal partnership
OIA: Office of Immigrant Affairs
